# Harmonized connectome resampling for variance in voxel sizes

**DOI:** 10.1016/j.mri.2025.110424

**Published:** 2025-05-19

**Authors:** Elyssa M. McMaster, Nancy R. Newlin, Gaurav Rudravaram, Adam M. Saunders, Aravind R. Krishnan, Lucas W. Remedios, Michael E. Kim, Hanliang Xu, Jongyeon Yoon, Derek B. Archer, Kurt G. Schilling, François Rheault, Laurie E. Cutting, Bennett A. Landman

**Affiliations:** a Department of Electrical and Computer Engineering, Vanderbilt University, Nashville, TN, USA; b Department of Computer Science, Vanderbilt University, Nashville, TN, USA; c Vanderbilt University Institute of Imaging Science, Vanderbilt University Medical Center, Nashville, TN, USA; d Department of Radiology and Radiological Sciences, Vanderbilt University Medical Center, Nashville, TN, USA; e Vanderbilt Memory and Alzheimer’s Center, Vanderbilt University School of Medicine, Nashville, TN, USA; f Department of Computer Science, Université de Sherbrooke, Sherbrooke, QC, Canada; g Vanderbilt Kennedy Center, Vanderbilt University, Nashville, TN, USA; h Peabody College of Education, Vanderbilt University, Nashville, TN, USA

**Keywords:** Connectomics, Tractography, Diffusion MRI, Harmonization, Spatial sampling

## Abstract

Diffusion MRI (dMRI) fiber tractography presents exciting opportunities to deepen our knowledge of human brain connectivity and discover novel alterations in white matter. To date, there has been no comprehensive study characterizing the effect of dMRI voxel resolution on the resulting connectome for subject data. We assessed the statistical significance of graph measures derived from dMRI data by comparing connectomes from the same scans across different resolutions with 44 subjects (32 female) from the Human Connectome Project – Young Adult dataset (HCP-YA) with scan/rescan data (88 scans). We explored 15 isotropic and anisotropic resolutions, generated tractography and connectomes, and compared graph measures between each resolution and its nearest larger and smaller resolutions. Nearly all pairwise comparisons yielded statistically significant differences in graph measures (*p* ≤ 0.05, Wilcoxon Sign-Rank Test). Upon up sampling the 14 down sampled resolutions in 0.5 mm increments, we observed mitigation of the spatial sampling effect on both the tractography and the connectome’s complex graph measures. To investigate translational impact, we resampled 22 subjects from HCP-YA to the resolutions of two major national studies and up-sampled this data back to 1 mm isotropic with different interpolation methods. Similarity in results improved with higher resolution, even after initial down-sampling. To ensure robust tractography and connectomes, resample data to 1 mm isotropic resolution.

## 1. Introduction

Diffusion weighted MRI (dMRI) provides a non-invasive solution to map the random thermal motion of water molecules within biological tissue. Diffusion MRI, in combination with diffusion tensor imaging (DTI) or fiber orientation distributions (FODs), allows us to trace virtual projections of white matter connectivity in a process called tractography [1]. In DTI, we compute a tensor to show the direction of water molecule diffusion and degree of fractional anisotropy (FA) in the voxel of interest [2]. A higher voxel resolution in an image with DTI produces more tensors and can mitigate partial volume effects, but the DTI representation is limited in regions of crossing fibers [3–5]. FODs can model multiple fibers in the same voxel space, but is similarly limited as a voxel-wise operation [4–6]. The map of the streamlines of connectivity with the brain, a tractogram, can be generated with either DTI or FODs [1]. The tractogram’s matrix representation, a connectome, weights the brain’s connections according to a metric of interest, such as number of streamlines, mean length of streamlines, or fractional anisotropy, and can be interpreted by complex graph measures (see Supplemental Material 1 for definitions of complex graph measures) [7].

Complex graph measures have been applied to many diseases and disorders, but with different image resolutions and other acquisition parameters, which leads to variability in results. Studies of tractography and connectivity suffer from variability in their results from sensitivity to brain parcellation [8], acquisition parameters such as angular and spatial resolution [9–11], scan-rescan variability [12], reconstruction [13], and the tractography algorithm [14]. [15] Studies have used complex graph measures to quantify white matter changes across age groups and conditions, with voxel resolution ranging from state-of-theart research scanners with high resolution acquisition resolution to clinical resolution data [16–18].

**Fig. 1.**
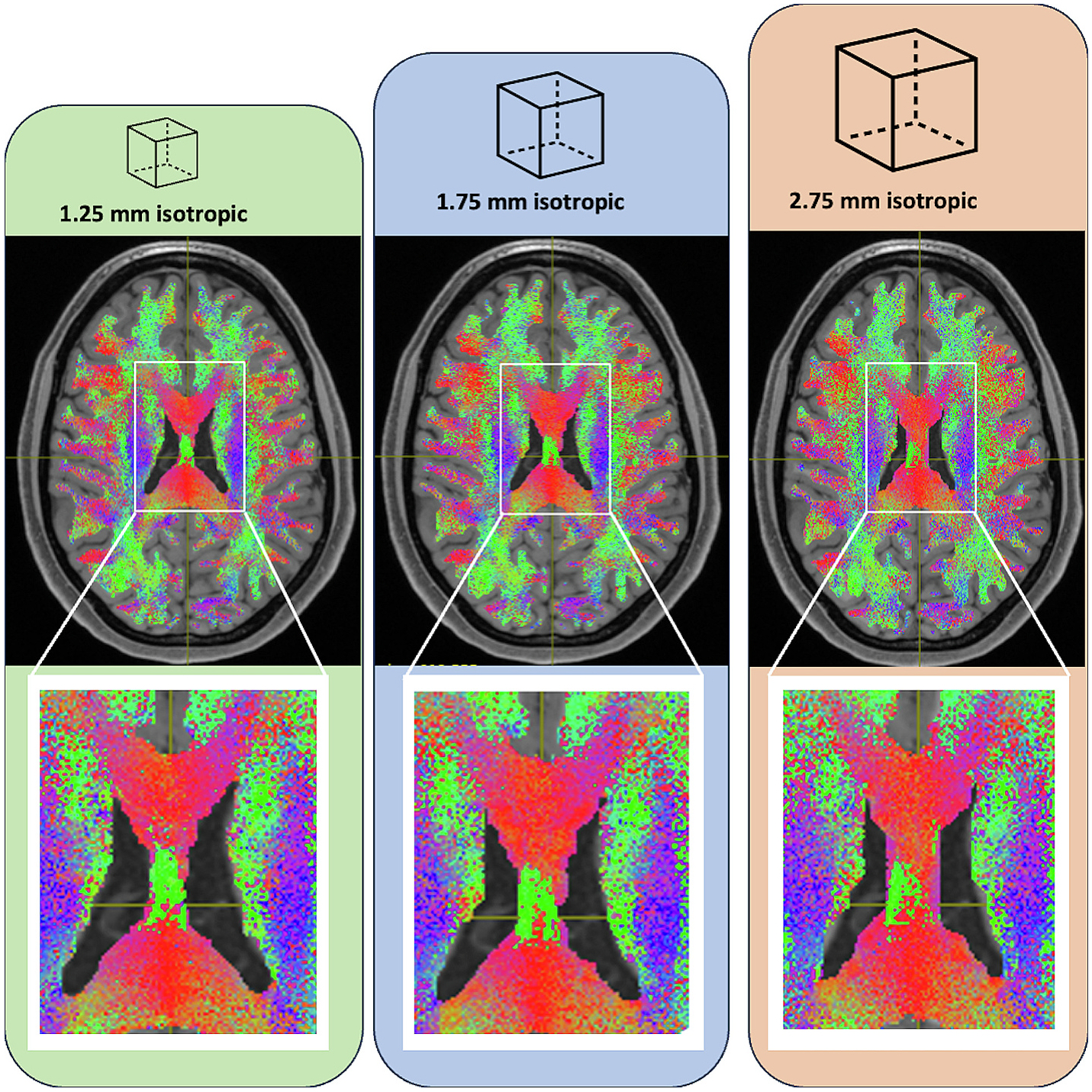
We visualize one subject at the acquisition resolution of 1.25 mm isotropic, and down sampled to 1.75 mm isotropic and 2.75 mm isotropic. Though the images show the same anatomy in the same acquistion, we observe thickening of the coprus callosum and the narrowing of the ventricles as the voxels increase in size.

The processes to generate these voxel-wise representations of the dMRI signal and human brain map are inherently tied to voxel resolution. The construction of a connectome begins with the generation of a tractogram. Tractography algorithms follow anisotropy within the microns of diffusion to delineate white matter from other tissue, but the parameters to do this, like ideal voxel size and tractography step size, have no gold standard [19,20]. A connectome, or connectivity matrix, uses the seed points from the tractogram as the nodes of the complex graph network. The lack of a generalized protocol for spatial resolution leads to a wide range of results in connectomes generated from tractography despite identical anatomy at different resolutions (Fig. 1). This inconsistency introduces a harmonization problem – how do we leverage the data we have to generate more reproducible and repeatable results?

Previous studies have sought to determine the sensitivity of connectomes to other factors, but none have shown an avenue for harmonization of subject data acquired at different resolutions. Zhan, et al. investigated the role of angular sampling in diffusion imaging measures [21]. Schilling, et al. studied the effect of scanner protocol effects on microstructure, including acquisition resolution from different scanners [22]. In 2012, Tournier et al. introduced MRTrix as a set of tools to analyze diffusion images. Based on the 2 mm isotropic and larger spatial resolution used in most dMRI studies and a reliance on visual inspection due to a lack of metrics at the time, spatial sampling showed little effect on the tractography process [5]. More recently, connectome adjacency matrices have become a popular representation of the tractography map because they quantify metrics that may not be obvious based on visual inspection [17,23–25]. A 2024 study by Zhong, et al. collected data from two different sites with different acquisition resolutions and concluded that voxel size has a larger impact on the variation between and across subjects than scan-rescan variability or coil differences [26], which invites an exciting opportunity to leverage the tools of the last decade to understand the effect of voxel size on connectomics in a way previously imperceptible to visual inspection.

Other studies have sought to perform spatial interpolation in DTI and tractography settings. Yang, et al. explored interpolation approaches to improve spatial resolution of low-quality data and evaluated based on fractional anisotropy and mean diffusivity in cardiac data. They establish a relationship between different levels of interpolation and a change in fiber generation and call for a similar study in brain data [27]. Coupé et al. proposed a super resolution method for diffusion weighted imaging that outperforms traditional methods like trilinear and B-spline interpolation and showed the potential of their method by illustrating fiber tracking quality in a deterministic tractography experiment with super-resolved images that decrease voxel dimensions from 1.2 mm isotropic to 0.6 mm isotropic and 0.4 mm isotropic [28]. With the growing interest in white matter modeling, super resolution in dMRI has taken off as a subject of interest in the last half-decade as the topic of the “Resolving to super resolution multi-dimensional diffusion imaging” (Super MUDI) Challenge at MICCAI 2021 [29]. Before implementing super-resolution techniques on dMRI datasets, we must establish if the common problems in connectome harmonization result from a lack of information that must solved with a super-resolution approach or a fixable issue in the tractography process. If we find that we can harmonize connectomes with basic up-sampling, we can bypass the computationally expensive step of a state-of-the-art AI super resolution approach.

In this work, we aim to demonstrate spatial harmonization in the context of tractography and connectomics. We propose a study to determine the degree to which the resolution of the diffusion-weighted image impacts complex graph measures. We aim to minimize the effect of acquisition resolution for more reproducible and repeatable maps of the brain’s connections. We evaluate the differences between connectomes with a pairwise comparisons between the graph measures at every resolution generated for this experiment via a Wilcoxon Signed-Rank test and compare the effect sizes between the relevant resolutions’ distributions of 12 complex graph measures with the Cohen’s *d* coefficient.

## 2. Methods

**Fig. 2.**
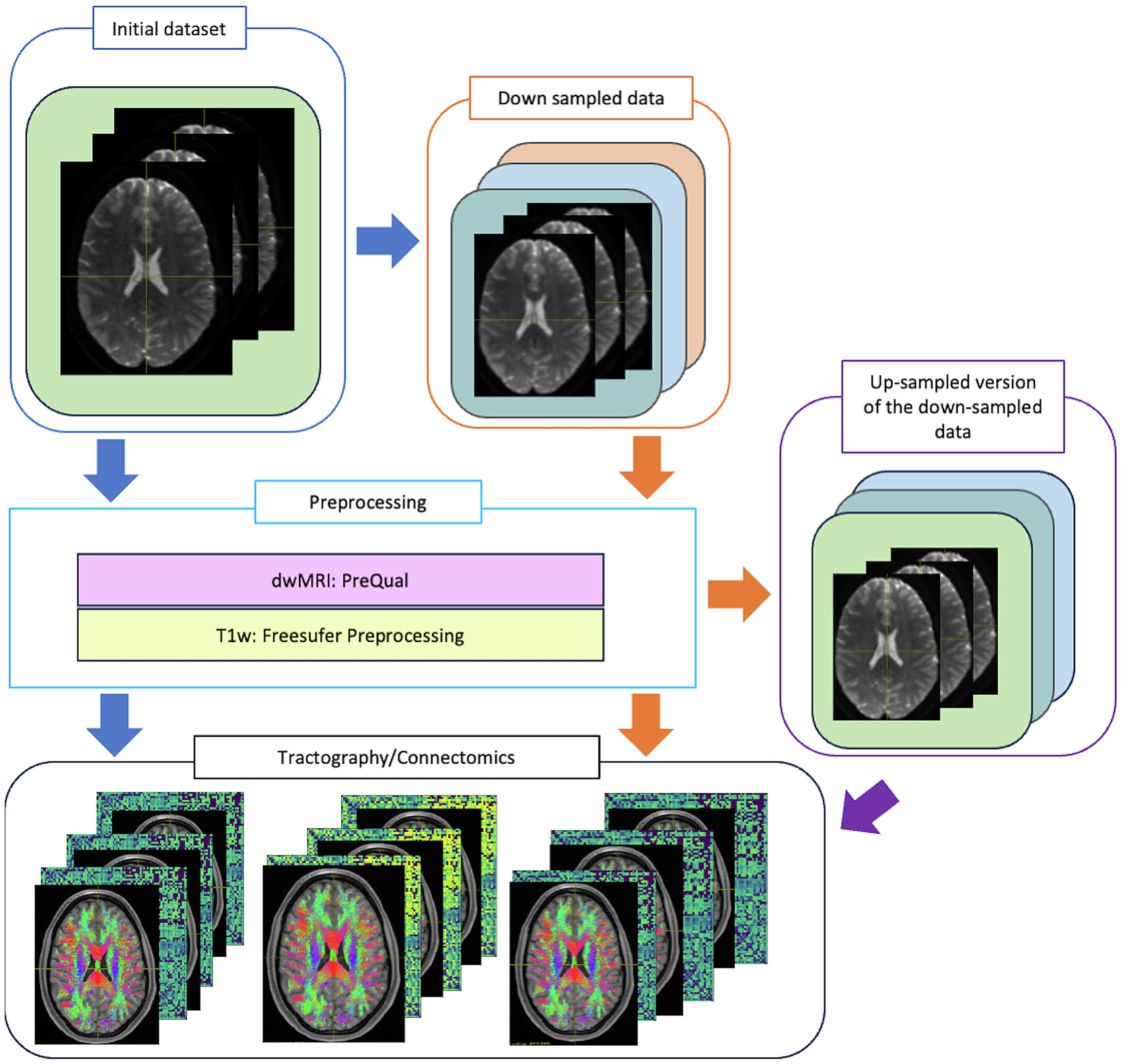
We designed the method to both understand the changes that happen between low- and high-resolution data. We preprocess the lower quality data and up sample it as though only lower quality data had been acquired. This way, we can understand the impact of information loss with lower spatial sampling.

We hypothesize that the resolution of a diffusion MRI image will introduce statistically significant changes in the complex graph measures of the resulting connectomes due to the interrelated nature of spatial resolution and white matter models. Additionally, we hypothesize that many of the connectome’s complex network measures become more reproducible with up sampling (Fig. 2). By regridding the image to a higher spatial resolution, we can address a common problem of tractography: multiple tissue types in a common voxel space [3]. We show that down sampling, even as little as 0.5 mm isotropic, fundamentally changes the integrity of the tractogram and the connectome because the tissue types in the voxels become more heterogenous as they increase in size. We explore the differences qualitatively with images of individual tractogaphy bundles.

### 2.1. Data

Our dataset consists of both dMRI and T1-weighted images 44 healthy subjects (32 female) of the scan/rescan data from the Human Connectome Project – Young Adult (HCP-YA) dataset. HCP’s protocol includes b-values 1000, 2000, and 3000 s/mm^2^ with a 3 T customized WU-Minn-Ox HCP scanner with a resolution of 1.25 × 1.25 × 1.25 mm^3^, with a minimal preprocessing pipeline on to correct eddy current distortions from the diffusion gradient and to align the diffusion images to harmonize all scan modalities [30]. Average age among subjects is 30.36 years with standard deviation ±3.34 [31].

### 2.2. Preprocessing

We use the PreQual preprocessing pipeline for efficient quality assurance, diffusion tensor analysis and visualization [32]. We use Freesurfer for the segmentation of the corresponding T1-weighted images for the connectome mapping and tractography bundle segmentations [33].

Once the initial HCP-YA dataset had been preprocessed with PreQual and Freesurfer, we use *mrgrid* from MRTrix3 to down sample the voxel sizes of each of the 44 scan/rescan subjects’ images [34]. We generated 14 additional datasets by increasing the voxel sizes of the images in 0.5 mm increments (1.25 mm isotropic to 4.75 mm isotropic and 1.25 × 1.25 × 4.75 anisotropic). In the case of down-sampling, *mrgrid* uses Gaussian smoothing as its default interpolation method, but maintains the image’s real-world coordinates. In the case of up-sampling, *mrgrid* defaults to cubic interpolation [34].

After preprocessing the images with lower spatial resolution, we simulated a study with preprocessed low-resolution data by reshaping the voxels of the lower quality images in 0.5 mm increments, isotropically and anisotropically. This allowed us to determine if the inconsistencies between low-quality and high-quality data are based on information loss or algorithmic choices because *mrgrid* only changes spatial dimensions, which introduces some degree of information loss [34].

### 2.3. Tractography

We generate tractograms with 10 million streamlines using MRTrix3’s default probabilistic tracking of second order integration over FODs, *tckgen* [23,34]. This default tracking places a seed point at every half-voxel [35], again tying the quality of the tractogram to the resolution of the diffusion image. For the tractography implementation, we limited seeding and a termination using the five-tissue-type mask and allowed backtracking [23,34,35]. We repeat the process with the same number of streamlines and conditions for the set of 88 HCP-YA DWI for 14 additional resolutions and the 14 resolutions of up-sampled lower resolution data.

### 2.4. Connectomics

We mapped the tractogram to a connectome with the Desikan-Killany atlas with 84 cortical parcellations from Freesurfer [23,33,36]. We generated connectomes weighted by number of streamlines, mean length of streamlines, and FA. We use the connectomes weighted by number of streamlines and mean length of streamlines to compute the complex graph measures [23,34]. We use 12 of the graph measures commonly used to analyze connectome organization [7,37] (see Supplemental Material 1 for the definitions of the complex graph measures included in our study). We use Scilpy to compute the complex graph measures with Python, as defined in the Brain Connectivity Toolbox (BCT) for Matlab [38]. The node-level computations are based on all the nodes in the network averaged together [23]. Although some graph measures are dependent on each other (for example, characteristic path length and global efficiency), we share many complex graph measures for as much transparency as possible. We acknowledge that some graph analysis studies control for network properties such as density [39], but the large voxel sizes introduced in this project exhibited such instability that the control would offer little consolation (see Supplemental Material 2). In a study with more voxel sizes in a closer range, this kind of analysis might offer more robust result.

### 2.5. Statistical analysis

We performed a Wilcoxon Sign-Rank test on the original resolution images and the down sampled images [40]. The null hypothesis asserts that the median differences between paired observations of changes in spatial resolution has no effect on the complex graph measures of a connectome, while the alternative hypothesis asserts that the differences between paired observations is not zero.

Once we rejected the null hypothesis, we up-sampled the down-sampled images to test our hypothesis about the potential to use spatial sampling for harmonization and used the Cohen’s *d* coefficient to measure the effect size between the original resolution images and those with some degree of information loss from the simple down sampling applied. We use the coefficients Cohen suggests to measure effect size: *d* = 0.2 indicates small effect size; *d* = 0.5 indicates medium effect size, and *d* = 0.8 indicates large effect size [41]. We aimed to quantify the effect size of the complex graph measures of the down-sampled change in resolution to the original HCP-YA data, and then up-sample the lower quality data and compare that to the original resolution as well. We used scan/rescan effect size as a baseline for variability in the complex graph measures in our result, and then we compared the first run of data between the resolutions.

## 3. Results

### 3.1. Quantitative results

The result of our test showed statistical significance between nearly every resolution pair in nearly every graph measure. Preliminary experiments showed that at the step between 2.75 mm isotropic and 3.25 mm isotropic, the complex graph measures begin to exhibit large Cohen’s d effect sizes between adjacent resolutions, and, qualitatively, spurious streamlines and large, unlocalized bundles. Tracts became physiologically implausible because of spatial sampling too large for a realistic human brain – this voxel resolution introduces multiple tissue types in the same space and the possibility of crossing fibers [3]. Since the voxels in major national studies do not exceed 3 mm isotropic, we decided to continue our project only regarding voxels smaller than that [31,42–44].

**Fig. 3.**
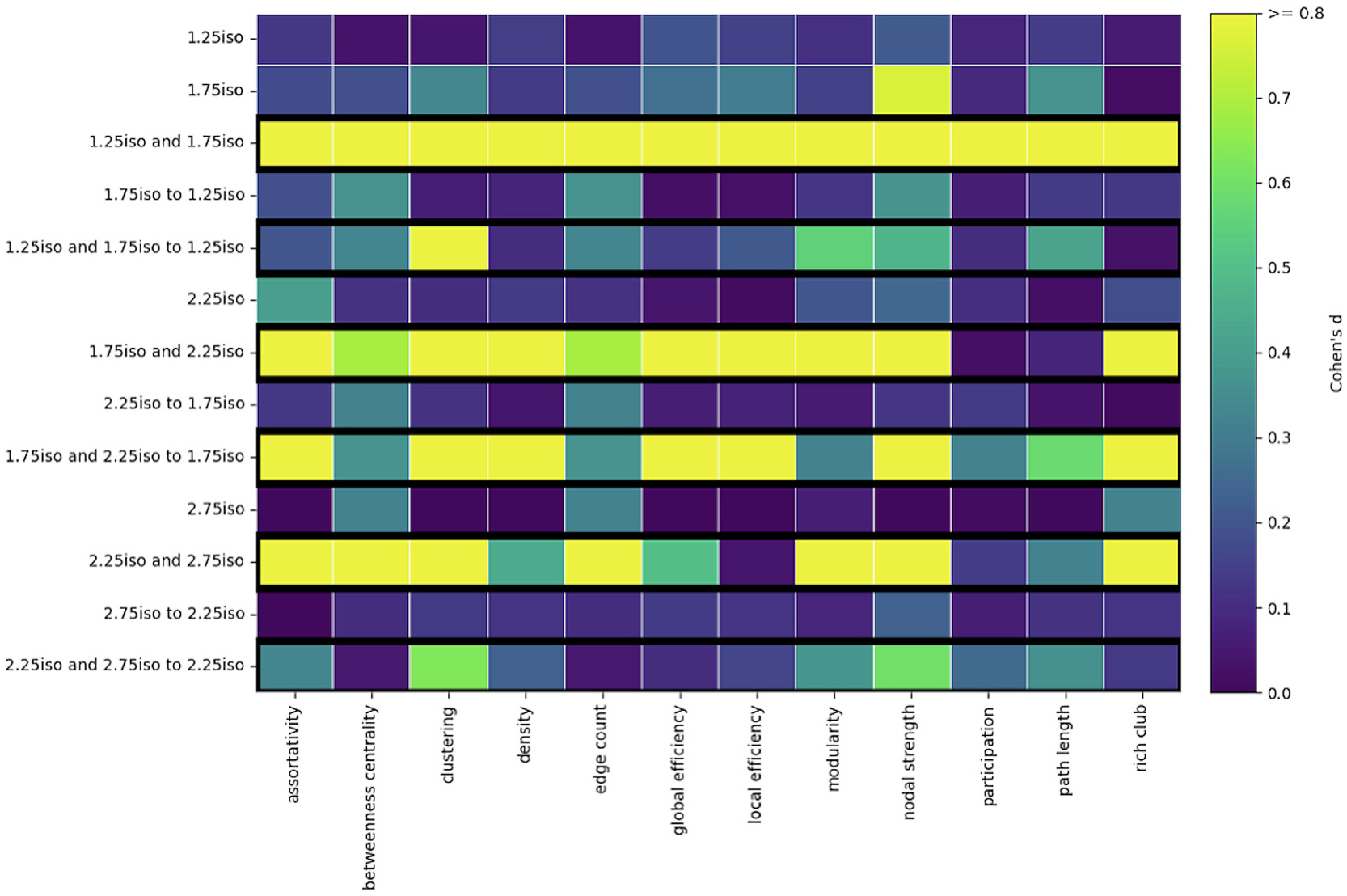
We use scan/rescan variability as a baseline to quantify the effect size inherent to the tractography process and then compared run 1 of different resoltuions to measure effect size between resolutions. We find that reshaping the voxels on lower quality data improves similarity with the high-quality data, whcih leads to a smaller effect size. The comparisons between run 1 of two different resoltuions are indicated with the bold outlined boxes.

The Cohen’s *d* effect size between the original dataset, low resolution data, and up-sampled versions of the down-sampled data indicates an algorithmic problem to overcome instead of an information loss problem across the complex graph measures. In Fig. 3, the rows with bold boarders represent the Cohen’s *d* coefficients between run 1 of different resolutions, while those not marked with boarders show scan-rescan variability within a single resolution. We observe a large Cohen’s *d* effect size when the data is down sampled by a factor of 0.5 mm, isotropic. When the same 1.75 mm isotropic data is up sampled to 1.25 mm isotropic, we find a smaller effect size on all measures except *clustering*.

### 3.2. Qualitative results

To interpret our qualitative results and to reaffirm our quantitative results, we resampled 22 subjects from the HCP-YA dataset and resampled the scan/rescan diffusion images to the resolutions with two major national studies: Alzheimer’s Disease Neuroimaging Initiative (ADNI3) [42,45], and The Baltimore Longitudinal Study of Aging (BLSA) [43,46] with cubic, nearest-neighbor, and sinc interpolation. By down-sampling our HCP data to the original resolutions of the major national studies, we can understand the efficacy of the simple down-sampling strategy for the harmonization of research data, even when we change interpolation method. We resampled our preprocessed 1.25 mm isotropic diffusion scan from HCP to 2 mm^3^ for ADNI3, and 0.8125 × 0.8125 × 2.18856 mm^3^ for BLSA with all three interpolation methods. We then took the down-sampled images from these two different resolutions and again up-sampled them to a common 1 mm isotropic, after which we performed the tractography steps again. We determined the Cohen’s *d* coefficient between the resolutions. We found a consistent large effect size between the original data and the resampled data (see Supplemental Material 3). Spatial sampling played a significant role in the generation of the streamlines and calculation of the complex graph measures, and with the much smaller standard deviation measurements we observe in the high-resolution data, we see great potential to harmonize connectomes with simple changes to spatial sampling regardless of interpolation.

**Fig. 4.**
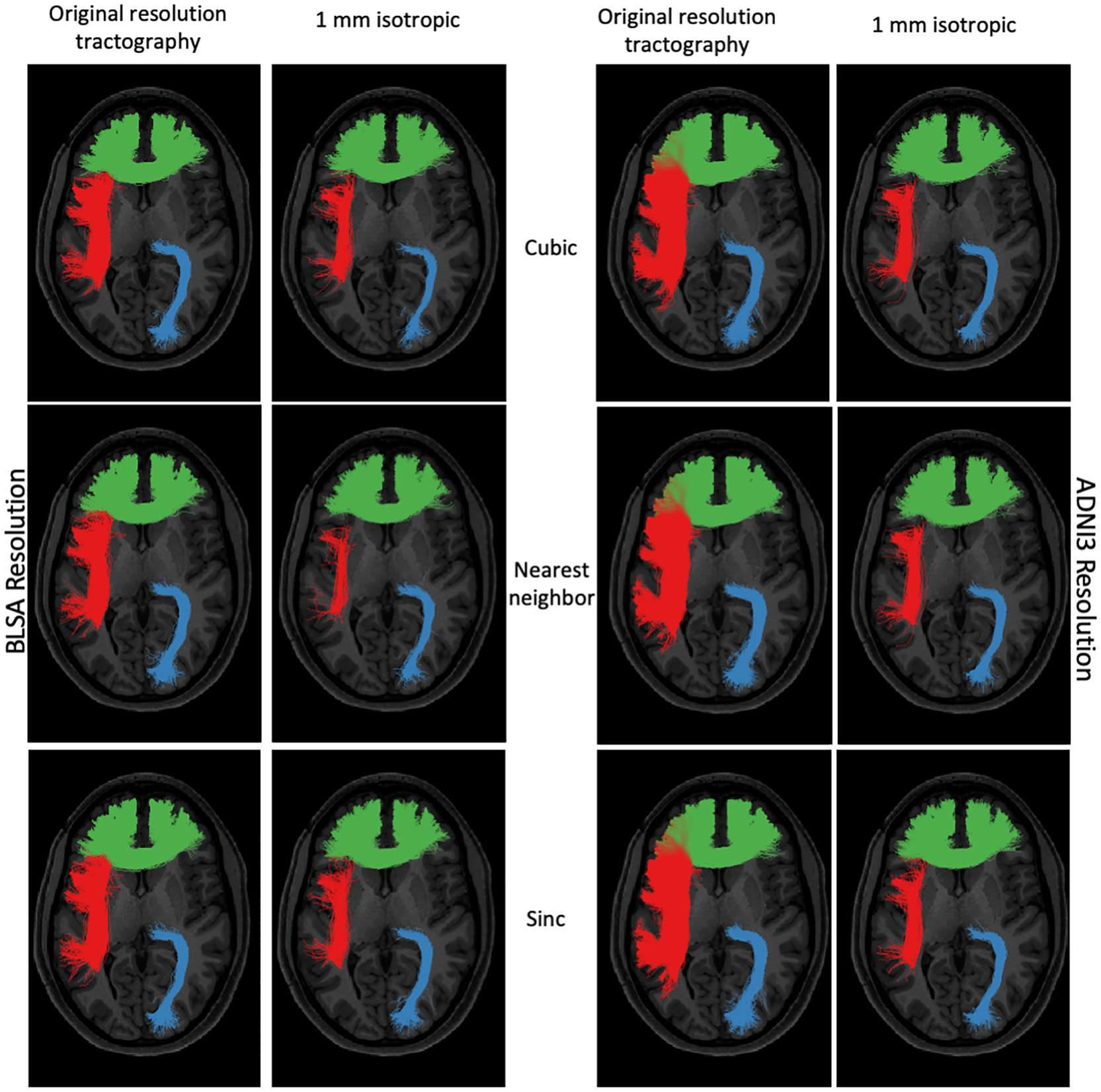
In the seame axial slice of a common T1, we visualize the left arcuate fasciculus, the corpus callosum, and the right optical radiation. We resample the HCP quality data to that of two major Alzheimer’s Disease studies and use cubic, nearest neighbor, and sinc interpolation to observe effects between interpolation choices. We obseve the effect of voxel size variation on the same subject with high curvature, loss of localization, and enlargement of tracts regardless of interpolation method. When we up-sample the image from the study’s resoltuin, the tractography appears more localized and anatomically plausible.

We use Recobundles to segment the corpus callosum, the left arcuate fasciculus, and the right optical radiation [47]. We overlay the white matter bundles over the common T1-weighted image for one subject to visualize the changes in the tracts based on the resolution of the diffusion image with OpenDIVE for the original dataset resolutions and the common resampled (1 mm isotropic) images (Fig. 4) [48]. The bundles from the major studies’ resolutions show several false-positive streamlines and areas of high curvature due to the inclusion of more tissue-types in a common voxel space. The bundles that result from the common 1 mm isotropic show a more concentrated body of streamlines directed toward a more specific region across interpolation methods. The outcome of this visualization shows consistency with the rest of our study: larger voxels and lower resolution results in less repeatable, less dense streamlines.

## 4. Discussion

We have investigated the implications of the inherent relationship between diffusion MRI, tractography, connectomics, and spatial sampling, and we have articulated an exciting path forward: spatial harmonization. We found a direct connection between spatial sampling and complex graph measure variability, and we have shown that simple up-sampling on both isotropic and anisotropic voxels to a common resolution lowers the variability of tractograms. By minimizing the possibility of multiple tissue types in a single voxel space while maintaining a resolution reasonable for computation, we can make the most out of the parameters of *tckgen* without making any changes to the algorithmic approach. Though we can change the tractogram’s step size, a change in step size does not mitigate the partial volume effects in voxel space that may impact the tractogram’s generation and is computationally expensive. This resampling strategy shows the potential for spatial sampling harmonization and indicates an exciting first step toward its large-scale study and implementation.

### 4.1. Limitations

Though many complex graph measures showed decreased variability from the simple up-sampling approach, the degrees to which these measures improved are not dependent on any single measure or resolution. Specifically, the *clustering* graph measure presents a unique challenge because its effect size remains large across resolution comparisons. It is an open question as to what is driving this effect and transitivity may offer an interesting perspective. *Clustering* is a local measure that may not be as stable in reproducibility studies compared to global metrics.

Since this study only used HCP-YA data, it limits the effect of spatial sampling on individuals with white matter diseases or disorders, and of different ages. Additionally, our dataset has a bias toward female scans. This study also did not account for site differences and acquisition parameters, as all data were collected by HCP with consistent acquisition parameters and high angular resolution. We believe that these variations may add additional complex graph measure variability that simple up-sampling may not be able to account for. We also acknowledge the possible biases in our connectome computation that could result from our choice of the relatively coarse Desikan-Killiany atlas [36]. Variations in graph measures may occur with an implementation of a finer parcellation [49]. We note that resampling higher than 1 mm isotropic may be computationally intensive for any downstream data processing.

We found statistical significance at every 0.5 mm step, isotropic and anisotropic, in the complex graph measures of the HCP-YA data and its lower-resolution counterparts. With simple up-sampling, we produce more reproducible and repeatable complex graph measures, even with the information loss inherent to down sampling. We have shown that this strategy is effective on resolutions used in major national studies on white matter diseases and disorders. Our recommendation is to up sample diffusion data to 1 mm isotropic before tractography to minimize effects of spatial sampling on the process.

## Supplementary Material

1

## Appendix A. Supplementary data

Supplementary data to this article can be found online at https://doi.org/10.1016/j.mri.2025.110424.
