# Supplementary material for: Harmonized connectome resampling for variance in voxel sizes
Source: Magn Reson Imaging. Author manuscript; Available in PMC 2026 Jun 9. (PMC13249084; doi:10.1016/j.mri.2025.110424)
Supplement: 1 [file NIHMS2176204-supplement-1.docx]

## Supplemental Material 1: Definition of Graph Measures

**Assortativity:** The extent to which nodes connect to nodes with a similar number of connections, and ranges from -1 to 1 (A negative indicates connections to with dissimilar numbers of connections, 0 shows no correlation, and a value close to 1 indicates connections to nodes with similar numbers of connections) [7], [32], [43].

**Betweenness Centrality:** The fraction of the shortest paths in the network that contain a given node. Ranges between 0 and 1 (A low value indicates a node with few shortest path, 0.99 indicates a node that lies on almost all shortest paths)[7][16], [32], [44].

**Clustering Coefficient:** The geometric mean of all triangles associated with a node [16], [32], [45].

**Density:** The fraction of present connections to all possible connections, ranges from 0 to 1 (A low value represents a sparse network when a high one indicates a densely populated network) [16], [32].

**Edge Count:** The number of connections in the characteristic path length. Ranges from 0 to positive infinity. [16].

**Global Efficiency:** Average inverse shortest path length. Ranges from 0 to 1, where a larger number indicates shorter paths and greater efficiency, but our implementation is weighted by shortest path lengths [7], [32], [46].

**Local Efficiency:** Global efficiency calculated over the neighborhood of a node. Ranges from 0 to 1, but our implementation is weighted by shortest path lengths [7], [32], [46].

**Modularity:** The ability to divide nodes in the network into distinct groups, and ranges from 0 to 1 (A low value indicates difficulty in separating the nodes into distinct groups while a high value indicates that the networks show a strong tendency to be separated into clusters) [7], [32], [47].

**Nodal Strength:** Sum of weights of links connected to a node [16], [32].

**Participation Coefficient:** Measures the variety of connections that nodes have to other nodes in different modules; degree to which a node interacts with other communities. Ranges from 0 to 1 (A low value shows low mixing between groups from different modules, a high value shows high mixing) [7], [32], [48].

**Path Length:** Number of edges traversed in the shortest path between nodes. Ranges from 0 to positive infinity [7], [32], [49].

**Rich Club:** Density between high-degree nodes. Ranges from 0 to 1 (A low value shows sparsely connected high-degree nodes and a low value shows densely connected nodes) [32].

## Supplemental Material 2: Controlling for Density

Characteristic Path Length vs Density


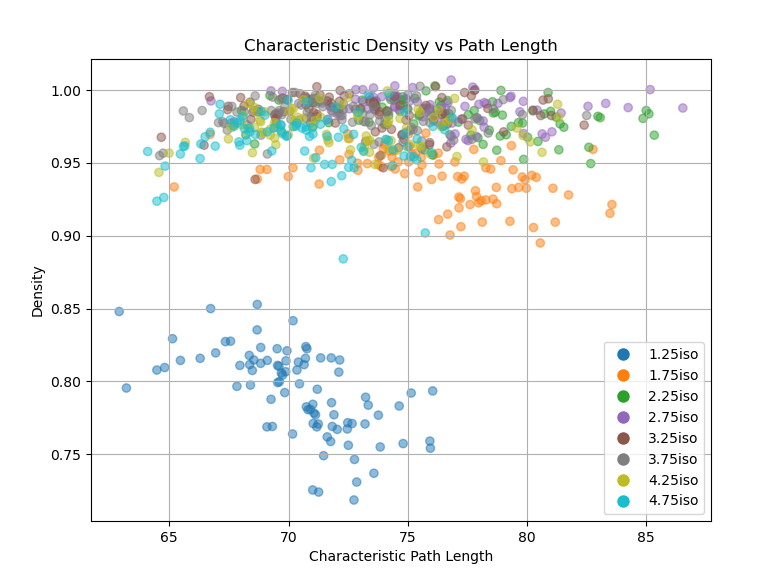


*Supplemental Material 2: We plotted characteristic path length and density to see if normalization by density would impact our results. We observe that our dataset would not benefit from normalization, but future studies on the effect of spatial resolution on complex graph measures may.*

Supplemental Material 3: Cohen’s *d* across interpolation methods


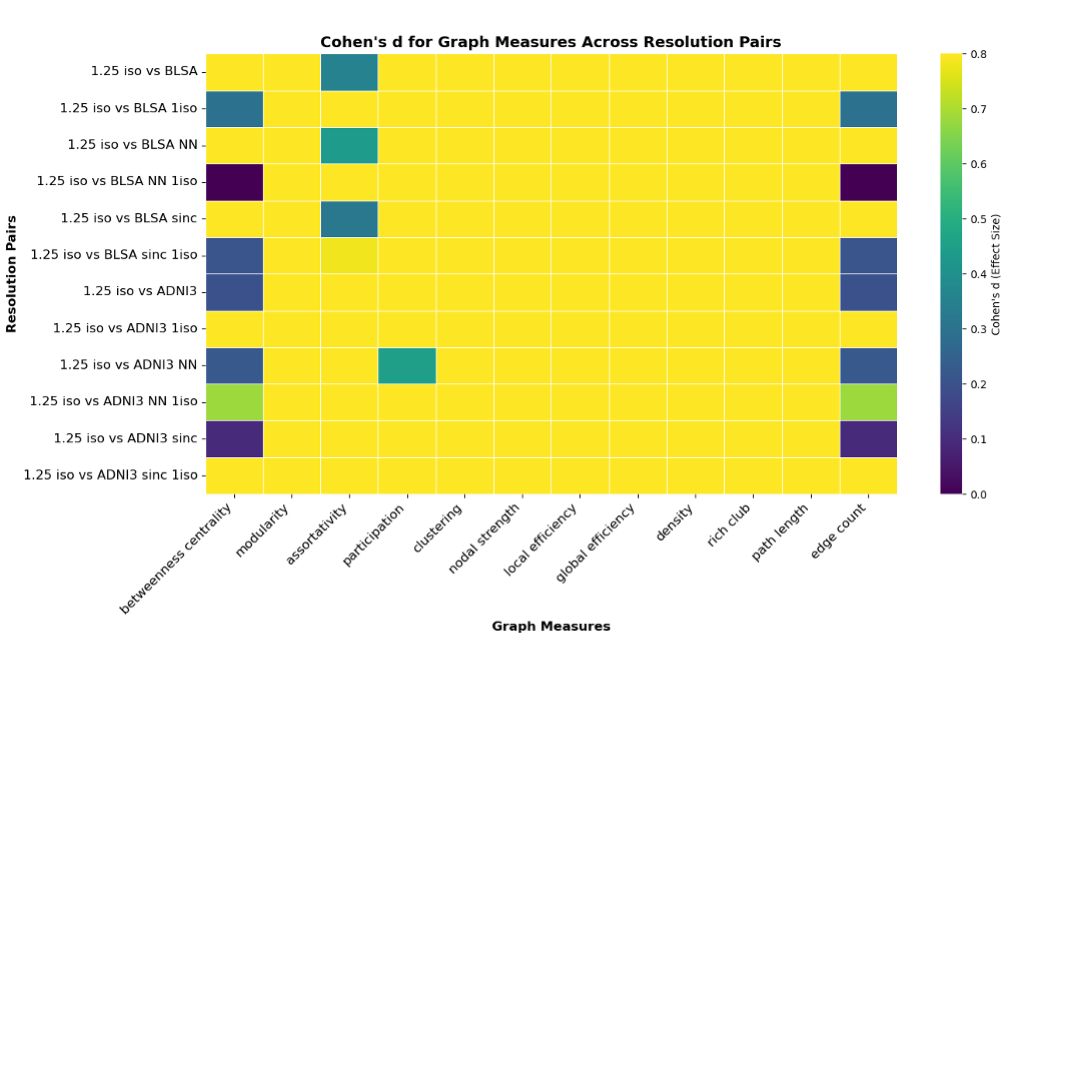


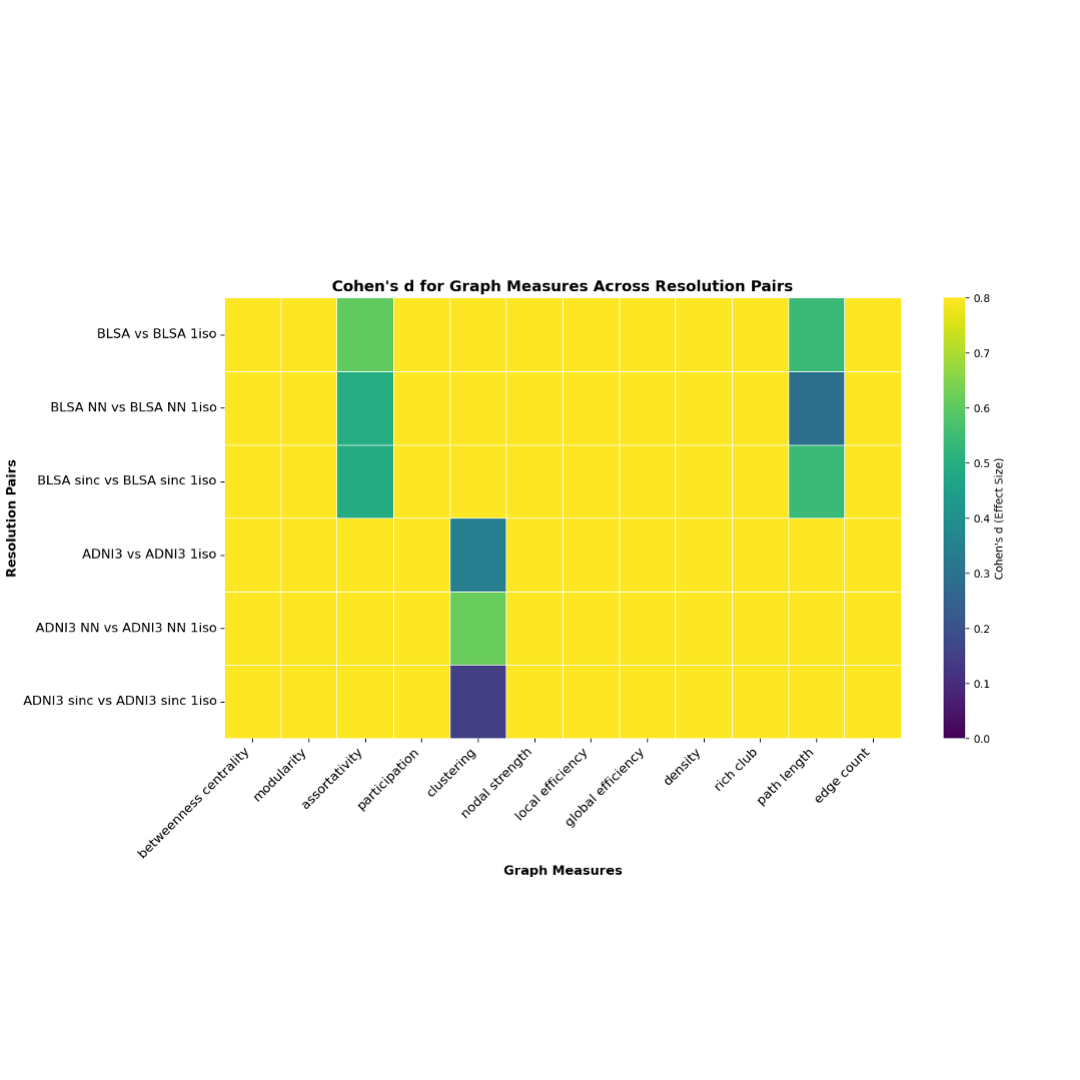


*Supplemental Material 3: We compute the Cohen’s d coefficient between the between the original resolution HCP-YA data, the data resampled to BLSA and ADNI3 resolutions and then resampled to 1mm isotropic. We can see large effect sizes between the resolutions even with different interpolation methods.*
